# Biodiversity Mapping in a Tropical West African Forest with Airborne Hyperspectral Data

**DOI:** 10.1371/journal.pone.0097910

**Published:** 2014-06-17

**Authors:** Gaia Vaglio Laurin, Jonathan Cheung-Wai Chan, Qi Chen, Jeremy A. Lindsell, David A. Coomes, Leila Guerriero, Fabio Del Frate, Franco Miglietta, Riccardo Valentini

**Affiliations:** 1 Impacts on Agriculture, Forest, and Natural Ecosystems Division, Euro-Mediterranean Center on Climate Change, Viterbo, Italy; 2 Foxlab, Fondazione Edmund Mach, S. Michele all' Adige, Italy; 3 Department of Geography, University of Hawai'i at Mānoa, Honolulu, Hawaii, United States of America; 4 The Royal Society for the Protection of Birds, Sandy, Bedfordshire, United Kingdom; 5 Department of Plant Sciences, University of Cambridge, Cambridge, United Kingdom; 6 Department of Civil Engineering and Computer Sciences Engineering, Tor Vergata University, Rome, Italy; 7 Department of Forest Resources and Environment, University of Tuscia, Viterbo, Italy; 8 A Rocha International, Cambridge, United Kingdom; 9 Institute of Biometeorology, National Research Council, Firenze, Italy; 10 Laboratory of Ecohydrology, Civil and Environmental Engineering, École Polytechnique Fédérale, Lausanne, Switzerland; 11 Department of Electronics and Informatics, Vrije Universiteit Brussel, Brussels, Belgium; 12 MountFor Project Centre European Forest Institute, Fondazione E.Mach, San Michele all' Adige, Italy; Clemson University, United States of America

## Abstract

Tropical forests are major repositories of biodiversity, but are fast disappearing as land is converted to agriculture. Decision-makers need to know which of the remaining forests to prioritize for conservation, but the only spatial information on forest biodiversity has, until recently, come from a sparse network of ground-based plots. Here we explore whether airborne hyperspectral imagery can be used to predict the alpha diversity of upper canopy trees in a West African forest. The abundance of tree species were collected from 64 plots (each 1250 m^2^ in size) within a Sierra Leonean national park, and Shannon-Wiener biodiversity indices were calculated. An airborne spectrometer measured reflectances of 186 bands in the visible and near-infrared spectral range at 1 m^2^ resolution. The standard deviations of these reflectance values and their first-order derivatives were calculated for each plot from the c. 1250 pixels of hyperspectral information within them. Shannon-Wiener indices were then predicted from these plot-based reflectance statistics using a machine-learning algorithm (Random Forest). The regression model fitted the data well (pseudo-R^2^ = 84.9%), and we show that standard deviations of green-band reflectances and infra-red region derivatives had the strongest explanatory powers. Our work shows that airborne hyperspectral sensing can be very effective at mapping canopy tree diversity, because its high spatial resolution allows within-plot heterogeneity in reflectance to be characterized, making it an effective tool for monitoring forest biodiversity over large geographic scales.

## Introduction

Mapping biological diversity is a fundamental conservation priority [1] as threats from habitat loss, fragmentation and climate change [2] continue to increase, and international agreements to reduce biodiversity loss (e.g. the Aichi Biodiversity Targets, CBD 2010) require a basis for prioritizing their response [3]. The need is particularly great for tropical forests, because they are major repositories of plant diversity [4], [5], [2]and play a critical role in the global carbon cycle and climate change mitigation, as recognized in international processes such as REDD [6], [7]. However, effective large-scale mapping of biodiversity in tropical forests has proven challenging and spatial information about tropical forest biodiversity is scarce.

Airborne and spaceborne sensors are able measure land cover characteristics over large scales so have the potential to map plant biodiversity at these required scales [8], [9], perhaps because spectral variation of reflectance values are correlated with spatial variation in the environment by means of landscape structure and complexity [10], [11]. The diversity of vegetation was found to relate to the NDVI [12], [13], [14], [15] and both the richness and evenness of tropical tree species were found to correlate with Landsat TM reflectance [16], [17] and spaceborne hyperspectral imagery [18]. Both the alpha and beta diversity of temperate deciduous forest could be predicted using ASTER imagery [19]. In more specific applications, multi-temporal data have been used to discriminate areas occupied by native trees from those dominated by invasive alien species [20], [21]. Given that canopy-tree diversity is often a good proxy for diversity of other taxonomic groups, remote sensing may have potential for mapping biodiversity in general [22]. Nonetheless, results using spaceborne sensors have so far shown only moderate to poor predictive power, even when using high resolution imagery [23], possibly due to low spatial and radiometric resolutions. Furthermore, most sensors on satellites are unable to capture fine-scale variation in biodiversity [16], [12], [23], [24].

Airborne hyperspectral sensors enable mapping at the fine scales desired by land managers [25], [26]. Hyperspectral data may provide information on how chemical and structural properties of vascular plants vary within and across ecosystems [27], [28] and technological improvements now allow them to be used to monitor terrestrial ecosystem characteristics [29], [30]. Hyperspectral data allow individual tree species to be identified from their signatures collected at the forest scale when using airborne sensors [31], [32], [33]. The addition of co-registered LiDAR data further improves performance by identifying intra and inter-canopy shadows which alter species signatures [34], [35]. [36] suggested that hyperspectral data reflects environmental conditions acting upon plants, such as soil pH, water availability, nitrogen availability and others, which are known to influence species distributions and community composition.

Few maps of tree biodiversity are available for Africa [37], [38], [39], [40], so our objective was to assess whether airborne hyperspectral data could be used for this purpose, focusing on canopy-tree biodiversity of a West African moist forest. We estimated alpha-diversity (Shannon-Wiener Index; [41]) in 64 permanently-marked plots in Gola National Park, Sierra Leone, which were also surveyed with a high-resolution airborne spectometer. Among the wide range of modeling tools, we selected Random Forests [42], a machine learning algorithm which handles high dimensional input with ease and has been demonstrated to function robustly [33], [36], [43]. In previous studies using hyperspectral data, RF has been used by [33] to discriminate tropical tree species and by [36] to analyze the species richness of a temperate montane forest in Germany.

## Materials and Methods

### 2.1 Study area and field data

**Figure 1:**
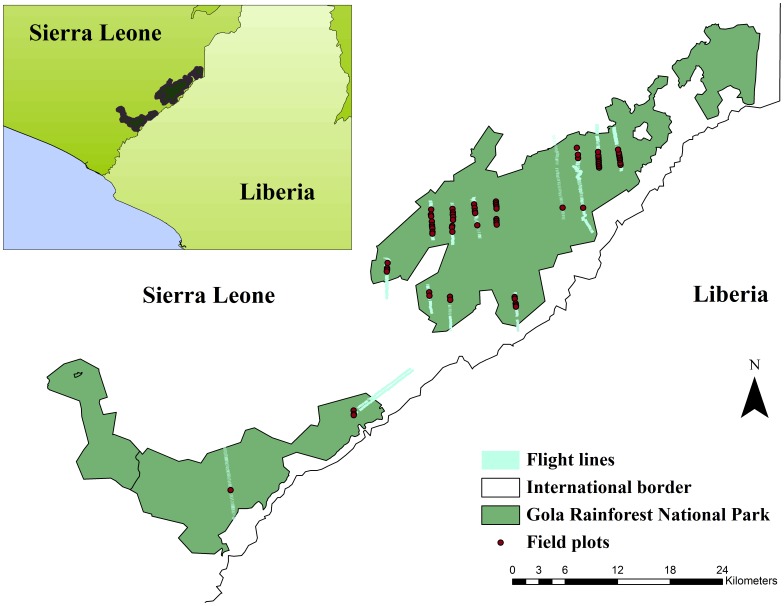
Gola Rainforest National Park, the study area in Sierra Leone. The strips of hyperspectral data which have been collected over the area are shown, together with the location of field plots overlapping the strips.

The study area is located at the westernmost end of the West African Upper Guinean Forest Belt, in Sierra Leone, covering the central portion of the Gola Rainforest National Park (GRNP) and some of the southern portion (Fig. 1), and included in an area defined by UTM coordinates (WGS84, 29N) N307591, E858452 (northeast) and N253197, E807411(southwest). GRPN is collaboratively managed by the Royal Society for Protection of Birds, the Conservation Society of Sierra Leone, and the Forestry Division of the Government of Sierra Leone; they provided the permits to collect the field data used in this study, and to fly over the area during airborne data acquisition. Field study did not involve endangered or protected species.

The region is characterized by lowland moist evergreen forests, with some drier types in place, dominated by Fabaceae, Euphorbiaceae and Sterculiaceae families [44]. The GRNP area has been protected through conservation programs since 1989 but commercial logging, most intensively in the southern block, was carried out in 1963–1965 and 1975–1989. Recent land cover mapping highlighted the importance of the GRNP in conserving this forest from anthropogenic pressure in the surrounding areas [45]. The climate is moist tropical, with annual rainfall around 2500–3000 mm, a dry season from November to April coincident with leaf-off condition of some semi-deciduous tree species, and an altitude of 70–410 m. Floristic information has been derived from a field survey carried out in 2006–2007 [46]. During that survey all trees with a Diameter at Breast Height (DBH) >30 cm were recorded in circular plots sized 0.125 ha. We selected the plots surveyed by an hyperspectral airborne campaign, excluding those located less than 1 km from the park boundary and those affected by cloud shadow in the hyperspectral data, retaining a total of 64 ground truth plots.

The biodiversity of a particular group of organisms in a location can be quantified in terms of richness and evenness [47]. An abundance-based measurement of plant diversity, like the Shannon-Wiener Index, should reflect the structural variability of a landscape much better than species richness, because it captures differences in composition and dominance structure of a given plant community [16]. We calculated the Shannon-Wiener index for each plot, according to the formula:
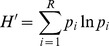
where *p_i_* is the proportion of individuals belonging to the *i*th species in the plot data (R =  total number of species).

### 2.2 Hyperspectral data

**Figure 2:**
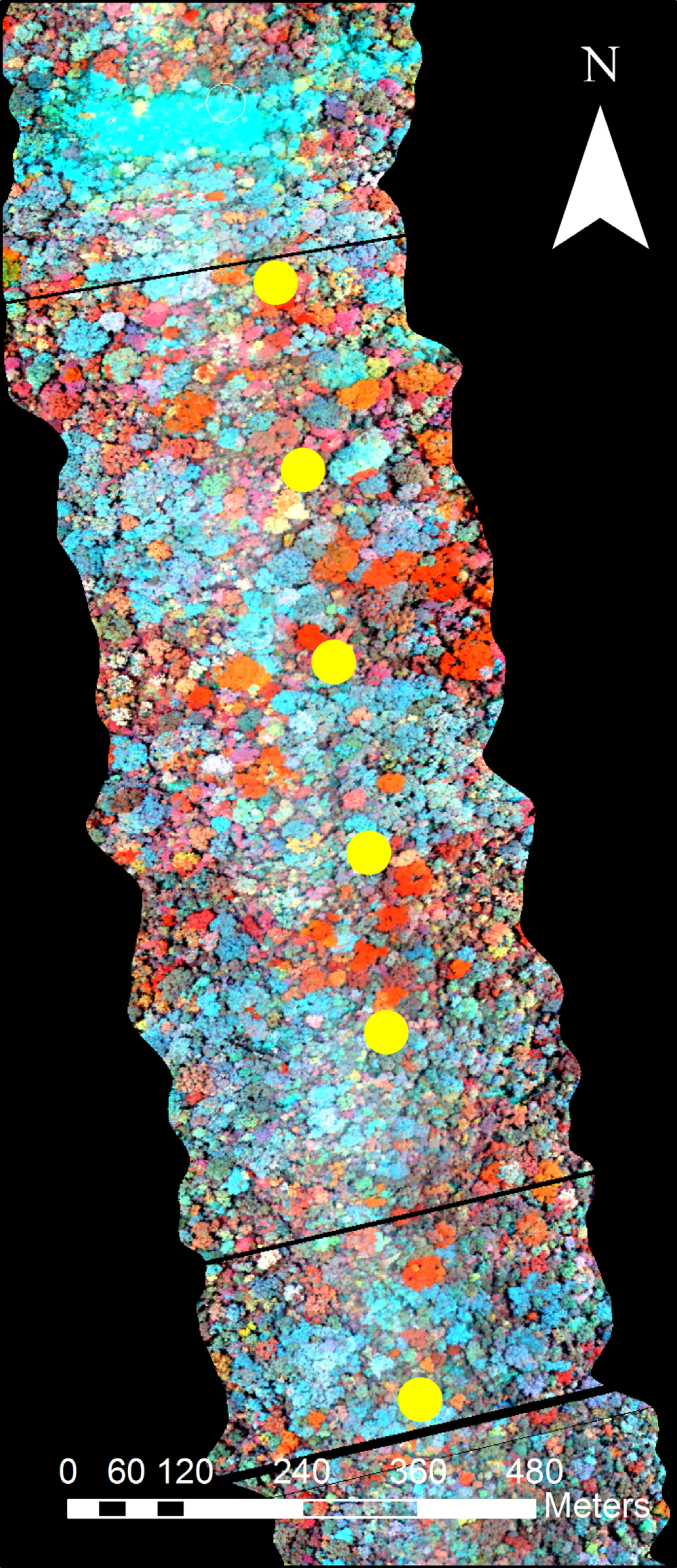
A strip of hyperspectral data (in false-color composite at 807.5 (R), 597.3 (G) and 467.3 (B) nm) showed as an example of available imagery, and with overlapped field plots areas, colored in yellow.

In March 2012 an airborne survey collected hyperspectral data over parts of the Gola GRNP, using an AISA Eagle sensor with FOV equal to 39.7°, set to record 244 bands with 2.3 nm spectral resolution in the 400–1000 nm range and spatial resolution of 1 m after radiometric correction and orthorectification (Fig. 2). Atmospheric correction of the hyperspectral image strips was performed using the Fast Line-of-Sight Atmospheric Analysis of Spectral Hypercubes (FLAASH) algorithm [48]. Due to high noise levels, all the bands out of the 450–900 nm range and four bands in the 759–766 nm range were removed, reducing the total number of bands to 186. Minimum Noise Fraction (MNF) transformation [49] was used to reduce noise further in the dataset. For each image strip, 9 to 15 MNF components were selected by visual screening and used to compute the inverse MNF and to transform the whole set of bands back to the original data space.

For each of the 0.125-hectare permanent plots, we extracted hyperspectral information from about 1250 pixels, and summarise these data in three ways: (a) the minimum, maximum, mean, and standard deviation of reflectances were calculated for the 186 hyperspectral bands remaining after data cleaning (n = 744; 186 bands ×4 metrics); (b) first-order derivatives of the hyperspectral reflectance curves can be useful for data analysis, as they allow small variations of the spectral curve to be enhanced and background noise to be suppressed [50], [51], so these were generated by dividing the difference between successive reflectance values by the wavelength interval, and then applying a seven-point moving filter to smooth results [52], [53]; we calculated the minimum, maximum, mean, and standard deviation of the derivative values obtained for each plot (n = 716; 179 derivatives × four metrics); and (c) we calculated the Photochemical Reflectance Index [54], the Red Edge Normalized Difference Vegetation Index [55], the Atmospherically Resistant Vegetation Index [56], the Vogelmann Red Edge Index [57], the Red Green Ratio [58], the Simple Ratio [59], and the Anthocyanin Reflectance Index [60]. We refer to these three datasets as (a) reflectance-based metrics, (b) derivative-based metrics and (c) vegetation indices.

### 2.3 Random Forests regression

We predicted the Shannon diversity index from spectral information contained in the three alternative datasets using Random Forests (RF), a machine learning algorithm employed in many different application domains [61], [42]. RF is a tree-based ensemble algorithm that generates hundreds or even thousands of alternative models (hence, ‘forests’). In building a tree, instead of using the best split among all variables, the best split among a subset of randomly chosen variables is used (hence ‘Random’). To incorporate the results from the hundreds of models, RF regression uses averaging. The importance of “features” (i.e. explanatory variables) can be ranked in two ways. The first is the increase in OOB-MSE if a particular feature is removed. The second is the increase of purity among the splitting groups in the process of building a decision tree if a particular feature is used. We chose to use the first strategy to understand the relative importance of different spectral regions in correlating with biodiversity.

RF was selected after careful consideration of its advantages and shortcomings. An advantage of RF is that it only has two parameters to tune - the number of random features for each split (*mtry*), and the number of the trees/models to build (*ntree*) – and having few parameters makes the result highly repeatable. Unlike some other tools, there is no assumption on data distribution. The embedded Out-of-Bag (OOB) strategy which separates one-third of the samples aside for evaluation each time when a model is built provides unbiased internal error estimation, and makes cross-validation unnecessary [61] http://www.stat.berkeley.edu/~breiman/RandomForests/cc_home.htm#ooberr). The OBB strategy also makes feature (i.e. explanatory variable) ranking very straightforward. In our data set, there are only 64 plots, which represent a relatively small sample size considering the great variety of tree species and the vast areal coverage of the study area. Thus a tool using internal estimates is well-suited. However, RF does have some well-recognised limitations. Given that it is a non-linear statistical modelling approach based on empirical data, models derived in one study region cannot be generalized to any new data sets. Additionally, different airborne data acquisition characteristics and preprocessing steps such as atmospheric and radiometric corrections further complicates a direct reuse of certain model. We chose RF after careful consideration, but do not claim it is necessarily the best tool, nor has comparison been made with other regression methods to show that RF provides the most accurate results.

RF was implemented within the R statistics framework (*randomForest* package; [62]) using procedures followed in numerous other studies [63], [64]. [42] suggests *mtry* should be set at 1/3 of the number of input features, while *ntree* should not normally exceed 1000 [61]. We varied *mtry* but found 1/3 was a good setting, and varied *ntree* between 100 and 1000 before settling on 200 after examining the goodness-of-fit statistics. RF regression provides an estimate of the mean squared error of residuals, but this is calculated from the OOB strategy so is different from the MSE generated by least-squares regression. For this reason we call it OOB-MSE. We calculated a pseudo-R^2^ which is equal to 1- (OOB-MSE/% variability explained). Again, pseudo-R^2^ is indicative, and cannot be compared directly with conventional R^2^.

## Results

### 3.1 Forest plot data

**Figure 3:**
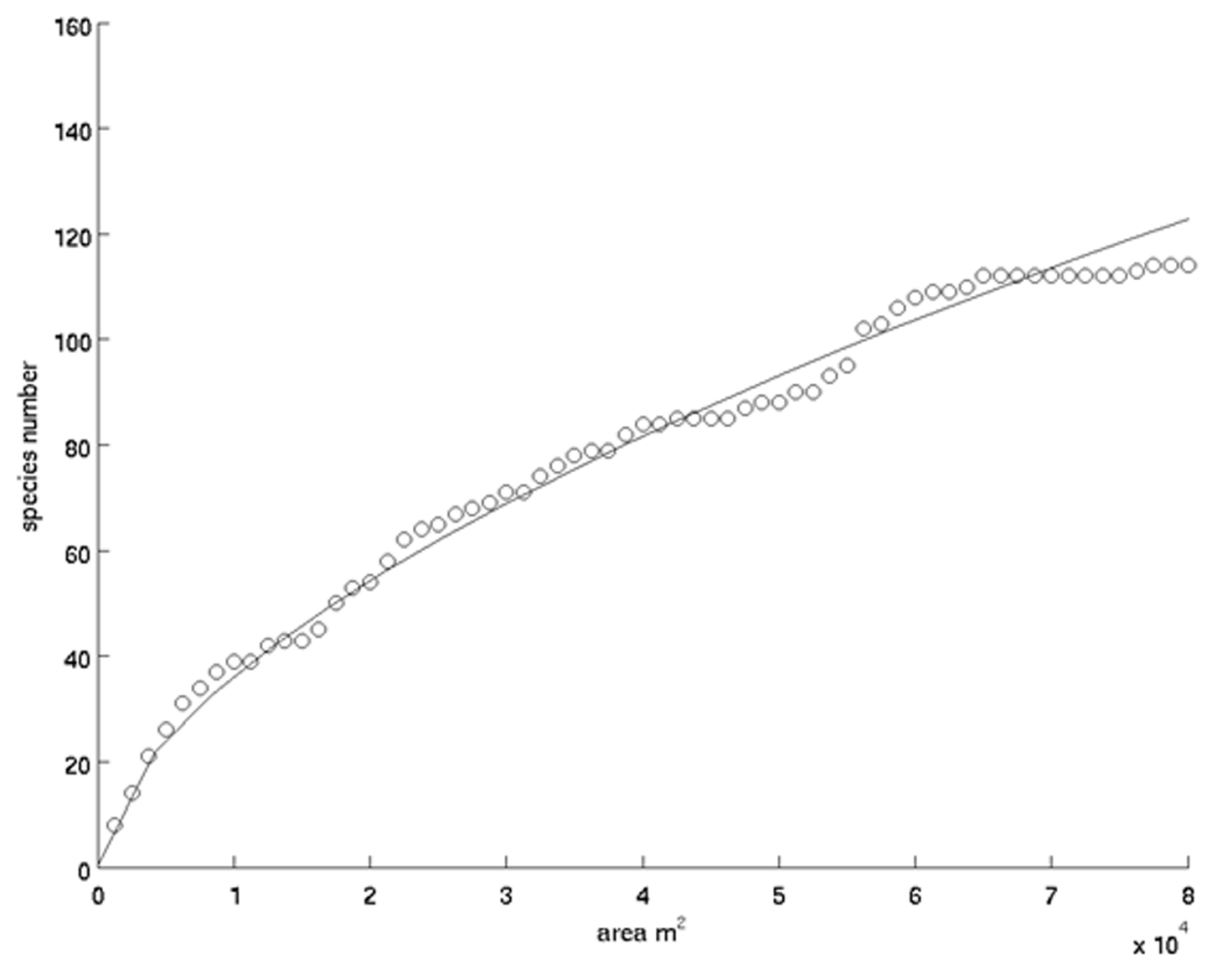
Species-area curve obtained from field data, illustrating the increase in species numbers resulting from the increase in the area of field data collection.

The 64 plots contained a total of 133 species. In the cumulated sampled area (8.125 ha) the total number of recorded trees was 676. The 15 most common species (i.e. >10 individuals) comprised >50% of individuals (Table 1), with Caesalpinioideae being the most represented sub-family. The species-area curve showed that the sampled area was big enough to capture most of the large-tree diversity of the site [65] (Fig. 3). The Shannon-Wiener index ranged between 0 and 2.63, with a mean value of 1.68 and a standard deviation of 0.48.

**Table 1:** List of most common trees (by individuals' number and % over all trees) found in the study area.

Species name	# of trees	% of trees	Family name
*Heritiera utilis*	61	9.0	*Malvaceae*
*Protomegabaria stapfiana*	57	8.4	*Phyllanthaceae*
*Cynometra leonensis*	50	7.4	*Caesalpinioideae*
*Brachystegia leonensis*	28	4.1	*Caesalpinioideae*
*Gilbertiodendron bilineatum*	28	4.1	*Caesalpinioideae*
*Stachyothyrsus stapfiana*	24	3.6	*Caesalpinioideae*
*Phyllocosmus africanus*	20	3.0	*Ixonanthaceae*
*Xylopia quintasii*	18	2.7	*Annonaceae*
*Parinari excelsa*	18	2.7	*Chrysobalanaceae*
*Calpocalyx brevibracteatus*	16	2.4	*Mimosoideae*
*Sacoglottis gabonensis*	14	2.1	*Humiriaceae*
*Octoknema borealis*	13	1.9	*Olacaceae*
*Uapaca guineensis*	13	1.9	*Euphorbiaceae*
*Berlinia confusa*	10	1.5	*Caesalpinioideae*
*Bussea occidentalis*	10	1.5	*Caesalpinioideae*
**Total**	**380**	**56.2**	

### 3.2 Regression results

**Figure 4:**
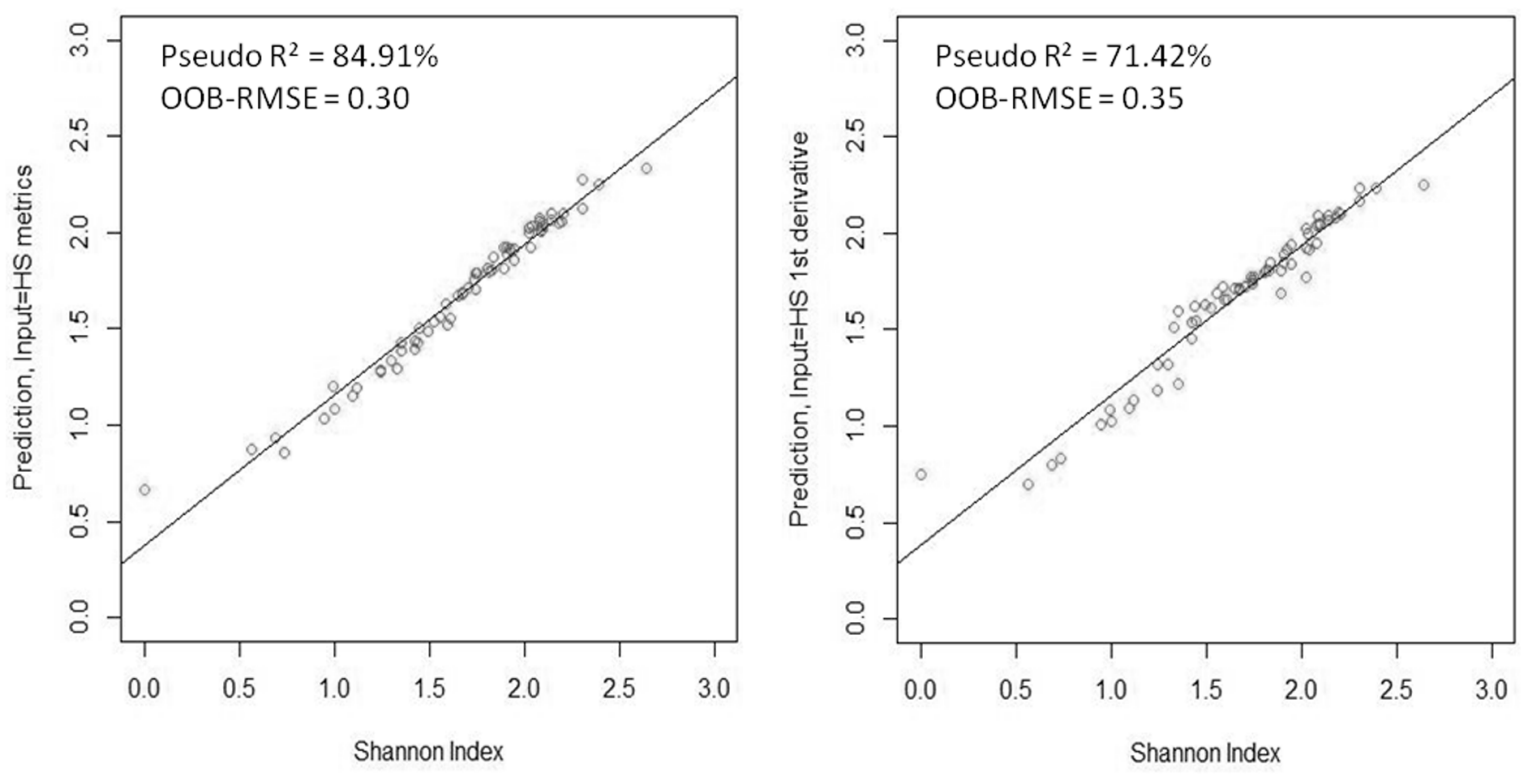
Scatterplots of the predicted versus the expected Shannon-Wiener index values, obtained by two models, on the left the one based on hyperspectral reflectance band metrics, and on the right the model based on first-derivatives reflectance metrics.

RF indicate that the Shannon-Wiener index can be predicted to a good level of accuracy using the plot-level statistics derived from hyperspectral bands (Figure 4 and Table 2). Models fitted using the reflectance-based metrics (i.e. calculated directly from the hyperspectral reflectances) had pseudo-R^2^ = 84.9% and OOB-RMSE  = 0.30. Models fitted using derivative-based metrics had lower explanatory power, with pseudo-R^2^ = 71.4% and OOB-RMSE  = 0.35. Vegetation indices were very poor predictors of diversity, giving rise to negative pseudo-R^2^ that indicate an inability of the models (on average) to explain any of the variability in biodiversity among plots The *mtry* and *ntree* for the HS metrics were set at 340 and 200, respectively. The *mtry* and *ntree* for the HS 1^st^ derivatives were 280 and 200 respectively.

**Table 2:** Random Forests models results using the three input sets.

Random Forests Out-of-bag estimates	
	*Shannon index*
Hyperspectral band reflectance metrics	pseudo-R^2^ = 84.91%, OOB-RMSE = 0.30
First derivatives reflectance metrics	pseudo-R^2^ = 71.42%, OOB-RMSE = 0.35
Vegetation indices	pseudo-R^2^: -15.97%, OOB-RMSE = 0.51

**Figure 5:**
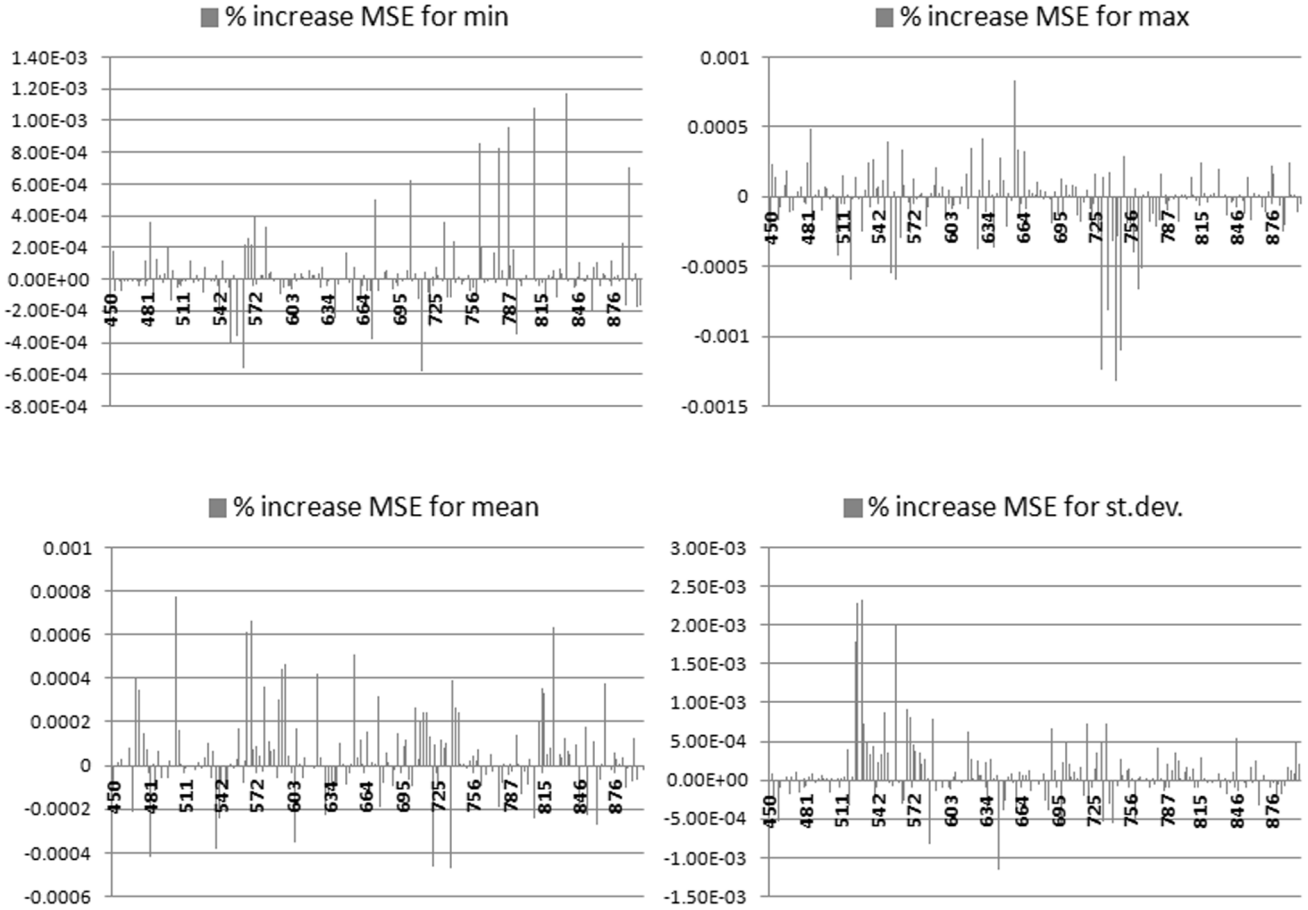
Ranking of hyperspectral metrics, a way to identify the regions most contributing to model success, with maximum, minimum, mean, standard deviation of band reflectance in the four different frames of the figure. The y-axis represents the percentage increase in OOB- MSE and the x-axis is the band region (in nm).

**Figure 6:**
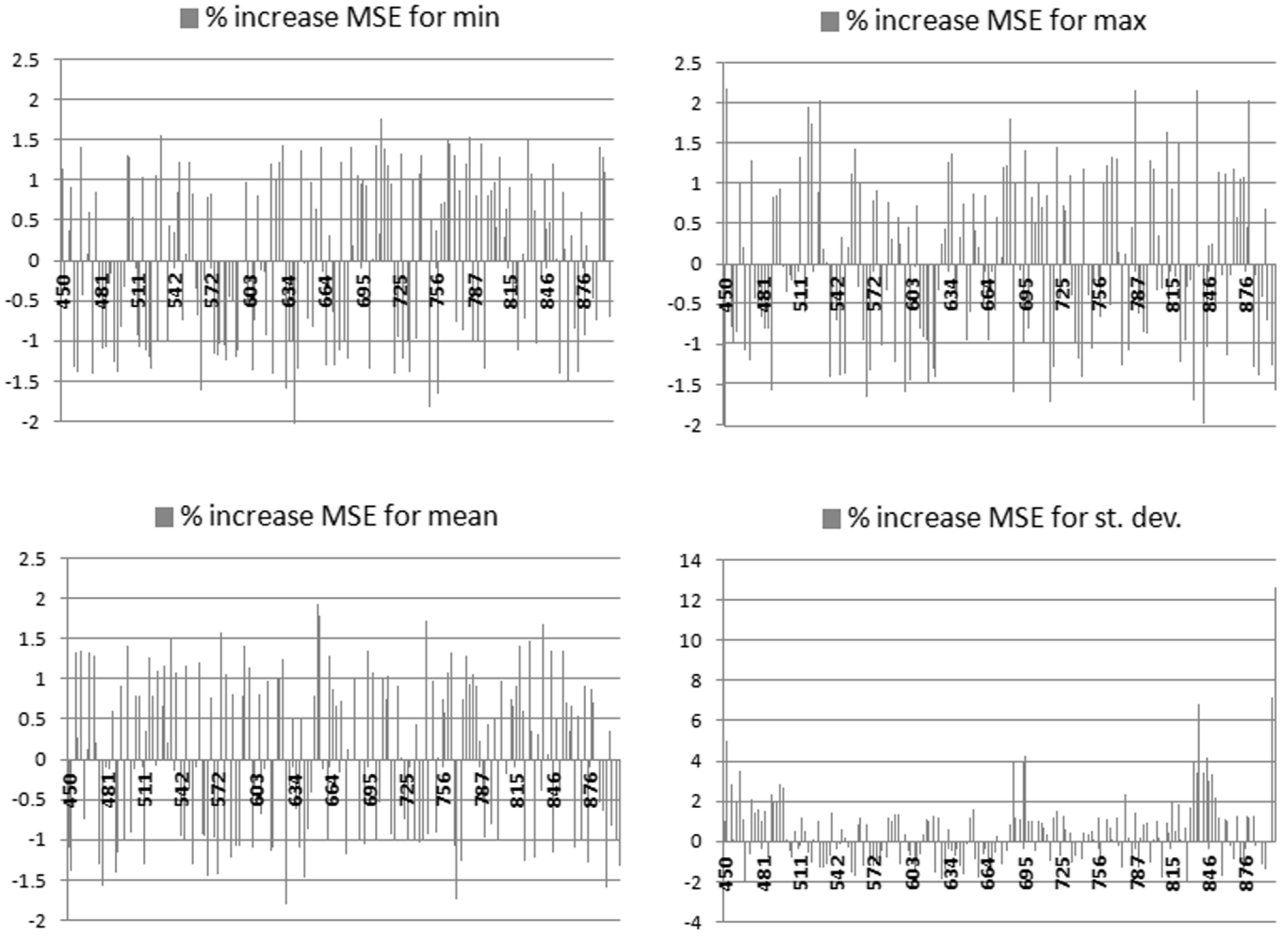
Ranking of derivative metrics, a way to identify the regions most contributing to model success, with maximum, minimum, mean, standard deviation of first derivatives of band reflectance in the four different frames of the figure. The y-axis represents the percentage in increase in MSE and the x-axis is the band region (in nm).

The rank importance of “features” (calculated from the percentage increase in OOB-MSE when features are removed one-by-one from the model) indicates that within-plot-variation in hyperspectral reflectances are strongly correlated with the biodiversity index. Fig. 5 shows the ranking of hyperspectral reflectance-based metrics (maximum, minimum, mean, standard deviation of band reflectance) and Fig. 6 for the same metrics derived from the derivative-based dataset. When hyperspectral band metrics were used, the most important inputs were standard deviations from the green region, but contributions came from across the spectrum and for other metrics. When the derivative-based dataset was used, standard deviations from the near infrared region provided by far the highest ranking inputs, possibly due to the ability of the derivatives to suppress background signals that are prevalent in this region. In both of these models, the most important statistical metric was standard deviation, indicating that within-plot spectral variation is most informative in explaining diversity variation.

## Discussion

The West African study is the latest in a series to shows that airborne imaging spectroscopy can be effective at mapping tree diversity, particularly when recorded at high resolution [26]. The Random Forests algorithm found that within-plot variability in various hues of green was closely related to biodiversity (pseudo-R^2^ = 84.9%). Spectral reflectance vary greatly within individual tree crowns, between tree crowns of the same species, and are influenced by viewing geometry soil characteristics, forest vigor and the presence of liana [31], [32], [66], [38], [67]. However, these statistical analyses seem to have picked up the same signal as the naked eye would – that species-rich plots have a greater number of subtly different canopy colors than species-poor plots.

It is likely that the high resolution of our imagery (1 m pixel size vs >5 m diameter for a typical tree crown) was important to characterizing variability in spectral reflectances. In another study using high resolution imagery, [25] related vascular plants species richness in lowland forest in Hawaii to hyperspectral data from NASA's Airborne Visible/Infrared Imaging Spectrometer (pixel size of 3.6 m). They found that a regression model using derivative reflectances in regions associated with upper-canopy pigments, water and nitrogen content had a high goodness of fit (R^2^ = 0.85). In contrast, [36] had less success with lower resolution imagery in German montane forests. Using HyMap hyperspectral imaging (VIS-SWIR with 7 m spatial resolution), they obtained a maximum R^2^ of only 0.29 between species richness and reflectances, even when full waveform lidar data were included in the model. A better fit was obtained by the same sensors, this time at 5 m pixel size, when mapping Shannon-Wiener index within a savanna ecosystem (R^2^ of 0.41; [9]).

Among the studies based on satellite data at lower spatial resolution, one successful result has been obtained by [19] who retrieved the Shannon index with a coefficient of determination of 0.61 in a temperate forest using ASTER data. Another study directly estimating the Shannon-Wiener index in a tropical forest was realized by [18] using the Hyperion sensor in Costa Rica with 30 m pixel size. Using wavelet decomposition followed by a stepwise regression they found that the Shannon index could be predicted with a R^2^ of 0.84; vegetation indices were not such good predictors as wavelet features. The selected bands were those from the shortwave infrared region and one from the visible region of the spectra (621 nm). Our results are very similar to those obtained by [18] with respect to the ecosystem under analysis, the ability to retrieve the Shannon-Wiener index, and the poor results obtained with vegetation indices.

Together, these satellite and airborne based results suggest that spatial resolution is not the main key to successful mapping of biodiversity, and additional studies targeting different ecosystems are needed to clarify the relative importance of spatial and spectral resolution.

Derivative analysis might not be optimal for our aims and data, resulting in a lower R^2^ (Fig. 4; Table 2), similarly to what has been found by [32] in a tropical tree classification study. A possible explanation can be attributed to the fact that derivative is very sensitive to noise in the original spectrum. The residual noise is emphasized in the derivative spectra and this may vary according to the pixel location on the tree crown. In addition, environmental or stress factors such as moisture content and leaf age introduce subtle variations in crown reflectance that are enhanced by differentiation. Consequently, spectral variation within crowns can be unnecessarily boosted in the derivative domain, interfering with the identification of differences amongst crowns.

Species richness and Shannon-Wiener index are both widely used as indices of diversity in the remote sensing literature, but we argue that the abundance weighted index is more valuable from an ecological perspective. [25] estimated species richness and Shannon-Wiener index in lowland Hawaii from AVIRIS, obtaining a better goodness-of-fit when using species richness. Similarly, [36] found that species richness was the better of the two response variables in terms of goodness-of-fit for German forests. The reason we recommend using the Shannon-Wiener index is that ecosystem processes, such as water balance and nutrient cycles, depends primarily on the functional characteristics of the most abundant species [68]. The Shannon-Wiener index is weighted in favor of abundant-species, making it more useful for relating spectral signals to local ecological processes. However, a key open question in biodiversity studies is whether information on canopy biodiversity can be a surrogate for sub-canopy biodiversity; with this respect is of interest the [34] research, which estimated the diversity of foliar chemicals within the canopy as a whole using hyperspectral data, and related this to faunal and floral distributions.

There is currently great interest in using airborne remote sensing to go one step further, and map individual canopy species in tropical forests [26]. Biophysical and functional attributes of forest canopies that can be used to distinguish among individual species [69], and help in understanding the relationships among spectral response, foliar biochemical components and canopy geometry. For instance, [20] used spaceborne hyperspectral data to map the spread of a nitrogen-fixing invasive trees in Hawaii, because the nitrogen-fixer was spectrally different from non-fixing trees. They also found that phenology is a key to distinguish species, and suggested the need for intense multi-temporal monitoring to maximize species separability. [70] have also discussed the role of hyperspectral remote sensing in tracking plant invasions, highlighting that these data can inform predictive models of invasions and species habitat suitability analysis. Using AVIRIS data from Hawaii, [71] found that differences in canopy spectral signatures were linked to differences measured in leaf pigment (chlorophyll, carotenoids), nutrient (N,P), and structural (specific leaf area, SLA) properties, as well as to canopy leaf area index. In a study addressing how leaf spectroscopy scales to canopy level reflectances, [72] used a leaf optical radiative transfer model (PROSPECT-5) to explore the relationship linking classification accuracy at the leaf level to canopy biodiversity, and found that it showed an asymptotic trend which suggests the uniqueness of spectral signature for a significant proportion of the 188 studied tropical species. Detecting individual species from aircraft is more technically demanding than the analyses presented here, but the approaches hold great promise and may eventually dispense off the need for diversity-index mapping.

## Conclusions

The present research demonstrates the ability of an airborne hyperspectral sensor to predict the canopy Shannon-Wiener index in African tropical forests, and is among those pioneer valuable efforts that could open the way to improved biodiversity monitoring. Airborne hyperspectral sensors represent today an important and cost-effective tool to target areas with high biodiversity, high vulnerability to change (e.g., occurring on deforestation fronts) and/or with tree species that are of particular importance [66].

However, data acquisition in remote and biodiversity rich study areas is still exceptionally challenging. Problems with data and ground truth gathering as those we faced, such as the time lag between field data collection and the airborne survey, or the difficulties in obtaining accurate geo-referencing of field plots, might affected the results and have to be carefully considered when planning hyperspectral-based biodiversity monitoring.

Our experience shows that the use of standard devation of reflectance provides satisfactory results, in agreement with the spectral variation hypothesis. We find RF an effective regression tool which is fairly easy to use, and the OOB feature ranking a valuable source of info pertaining to the feature importance.

Overall, considering other available studies and results, there is a clear need to further increase research on the use of airborne and spaceborne hyperspectral imagery in different ecosystems, to enhance our understanding of the optimal techniques to map the distribution of life on earth. This should be accompanied by quality biodiversity field information collected with proper sampling strategies. For future studies planning, the addition of SWIR spectral region should be considered, as well as of airborne laser scanner (ALS) data, recently reported as valuable source of information for biodiversity [73], [35], [74].

## References

[1] Gaston KJ (2000) Global patterns in biodiversity. Nature 405: 220–227. 10821282 10.1038/35012228

[2] Thomas CD, Cameron A, Green RE, Bakkenes M, Beaumont LJ, et al (2004) Extinction risk from climate change. Nature 427: 145–148. 14712274 10.1038/nature02121

[3] Balmford A, Whitten T (2003) Who should pay for tropical conservation, and how could the costs be met?. Oryx 37: 238–250.

[4] Chapin FS, Zavaleta ES, Eviner VT, Naylor RL, Vitousek PM, et al (2000) Consequences of changing biodiversity. Nature 405: 234–242. 10821284 10.1038/35012241

[5] Foody GM (2003) Remote sensing of tropical forest environments: towards the monitoring of environmental resources for sustainable development. International Journal of Remote Sensing 24: 4035–4046.

[6] Díaz S, Hector A, Wardle DA (2009) Biodiversity in forest carbon sequestration initiatives: not just a side benefit. Curr. Opin. Environ. Sustain 1: 55–60.

[7] Paoli GD, Wells PL, Meijaard E, Struebig MJ, Marshall AJ, et al (2010) Biodiversity Conservation in the REDD. Carbon Balance and Management 23: 5–7. 10.1186/1750-0680-5-7 PMC3002342 21092321

[8] Turner W, Spector S, Gardiner N, Fladeland M, Sterling E, et al (2003) Remote sensing for biodiversity science and conservation. Trends in ecology & evolution 18: 306–314.

[9] Oldeland J, Wesuls D, Rocchini D, Schmidt M, Jurgens N (2010) Does using species abundance data improve estimates of species diversity from remotely sensed spectral heterogeneity? Ecological Indicators 10: 390–396.

[10] Palmer MW, Earls PG, Hoagland BW, White PS, Wohlgemuth T (2002) Quantitative tools for perfecting species lists. Environmetrics 13: 121–137.

[11] Simonson WD, Allen HD, Coomes DA (2012) Use of an airborne lidar system to model plant species composition and diversity of Mediterranean oak forests. Conservation Biology 26: 840–850. 22731687 10.1111/j.1523-1739.2012.01869.x

[12] Gould W (2000) Remote Sensing of vegetation, plant species richness, and regional biodiversity hotspots. Ecological Applications 10: 1861–1870.

[13] Nagendra H (2001) Using remote sensing to assess biodiversity. International Journal of Remote Sensing 22: 2377–2400.

[14] Kerr JT, Ostrovsky M (2003) From space to species: ecological applications for remote sensing. Trends Ecol. Evol 18: 299–305.

[15] Leyequien E, Verrelst J, Slot M, Schaepmanstrub G, Heitkonig I, et al (2007) Capturing the fugitive: applying remote sensing to terrestrial animal distribution and diversity. Int. J. Appl. Earth Obs. Geoinform 9: 1–20.

[16] Foody GM, Cutler MEJ (2003) Tree biodiversity in protected and logged Bornean tropical rain forests and its measurement by satellite remote sensing. J. Biogeogr 30: 1053–1066.

[17] Foody GM, Cutler MEJ (2006) Mapping the species richness and composition of tropical forests from remotely sensed data with neural networks. Ecol. Model 195: 37–42.

[18] Kalacska M, Sanchez-Azofeifa GA, Rivard B, Caelli T, Peter White H, et al (2007) Ecological fingerprinting of ecosystem succession: Estimating secondary tropical dry forest structure and diversity using imaging spectroscopy, Remote Sensing of Environment. 108: 82–96.

[19] Feilhauer H, Schmidtlein S (2009) Mapping continuous fields of forest alpha and beta diversity. Applied Vegetation Science 12: 429–439.

[20] Somers B, Asner GP (2012) Hyperspectral time series analysis of native and invasive species in Hawaiian rainforests. Remote Sensing 4: 2510–2529.

[21] Somers B, Asner GP (2013) Multi-temporal hyperspectral mixture analysis and feature selection for invasive species mapping in rainforests. Remote Sensing of Environment 136: 14–27.

[22] Gentry AH (1988) Changes in plant community diversity and floristic composition on environmental and geographic gradients. Annals of the Missouri Botanical Garden 75: 1–34.

[23] Rocchini D, Chiarucci A, Loiselle SA (2004) Testing the spectral variation hypothesis by using satellite multispectral images. Acta Oecologica 26: 117–120.

[24] Rocchini D, Ricotta C, Chiarucci A (2007) Using remote sensing to assess plant species richness: the role of multispectral systems. Applied Vegetation Science 10: 325–332.

[25] Carlson KM, Asner GP, Hughes RF, Ostertag R, Martin RE (2007) Hyperspectral remote sensing of canopy biodiversity in Hawaiian lowland rainforests. Ecosystems 10: 536–549.

[26] Féret JB and Asner GP (2014) Mapping tropical forest canopy diversity using high-fidelity imaging spectroscopy. Ecological Applications in press. 10.1890/13-1824.1 29160652

[27] Martin ME, Aber JD (1997) High spectral resolution remote sensing of forest canopy lignin, nitrogen, and ecosystem processes. Ecological Applications 7: 431–443.

[28] Ustin SL, Roberts DA, Gamon JA, Asner GP, Green RO (2004) Using imaging spectroscopy to study ecosystem processes and properties. BioScience 54: 523–534.

[29] Kumar L, Schmidt K, Dury S, Skidmore A (2001) Imaging spectrometry and vegetation science. In Imaging Spectrometry: Basic Principles and Prospective Applications. Van der Meer FD and De Jong SM eds. pp.111–155. Kluwer Academic Publishers.

[30] Thenkabail PS, Enclona EA, Ashton MS, Van Der Meer B (2004) Accuracy assessments of hyperspectral waveband performance for vegetation analysis applications. Remote sensing of environment 91: 354–376.

[31] Clark DA, Roberts DA, Clark DB (2005) Hyperspectral discrimination of tropical rain forest tree species at leaf to crown scales. Remote Sensing of Environment 96: 375–398.

[32] Zhang J, Rivard B, Sanchez-Azofeifa A, Castroesau K (2006) Intra- and inter-class spectral variability of tropical tree species at La Selva, Costa Rica: implications for species identification using HYDICE imagery. Remote Sensing of Environment 105: 129–141.

[33] Clark ML, Roberts DA (2012) Species-level differences in hyperspectral metrics among tropical rainforest trees as determined by a tree-based classifier. Remote Sensing 4: 1820–1855.

[34] Asner GP, Knapp DE, Kennedy-Bowdoin T, Jones MO, Martin RE, et al (2008a) Invasive species detection in Hawaiian rainforests using airborne imaging spectroscopy and LiDAR. Remote Sensing of Environment 112: 1942–1955.

[35] Feret JB, Asner GP (2012) Semi-supervised methods to identify individual crowns of lowland tropical canopy species using imaging spectroscopy and LiDAR.0. Remote Sens 4: 2457–2476.

[36] Leutner BF, Reineking B, Müller J, Bachmann M, Beierkuhnlein C, et al (2012) Modelling forest α-diversity and floristic composition— on the added value of LiDAR plus hyperspectral remote sensing. Remote Sens 4: 2818–2845.

[37] Levin N, Shmida A, Levanoni O, Tamari H, Kark S (2007) Predicting mountain plant richness and rarity from space using satellite-derived vegetation indices. Divers. Distrib 13: 692–703.

[38] Nagendra H, Rocchini D (2008) High resolution satellite imagery for tropical biodiversity studies: the devil is in the detail. Biodiversity Conservation 17: 3431–3442.

[39] Jürgens N, Schmiedel U, Haarmeyer DH, Dengler J, Finckh M, et al (2012) The BIOTA Biodiversity Observatories in Africa—a standardized framework for large-scale environmental monitoring. Environmental monitoring and assessment 184: 655–678. 21448628 10.1007/s10661-011-1993-y

[40] Townsend AR, Asner GP, Cleveland CC (2008) The biogeochemical heterogeneity of tropical forests. Trends Ecol. Environ 43: 424–431. 10.1016/j.tree.2008.04.009 18582987

[41] Shannon CE (1948) A mathematical theory of communication. The Bell System Technical Journal 27: : 379–423 and 623–656.

[42] Breiman L (2001) Random Forests. Machine Learning 45: 5–32.

[43] Chan JC-W, Paelinckx D (2008) An evaluation of Random Forest and Adaboost tree-based ensemble classifications and spectral band selections for ecotope mapping using airborne hyperspectral imagery. Remote Sensing of Environment 112: 2999–3011.

[44] Cole NHA (1993) Floristic association in the Gola rain forests: a proposed biosphere reserve. Journal of Pure and Applied Science 2: 35–50.

[45] Vaglio Laurin G, Liesenberg V, Chen Q, Guerriero L, Del Frate F, et al (2013) Optical and SAR sensor synergies for forest and land cover mapping in a tropical site in West Africa. International Journal of Applied Earth Observation and Geoinformation 21: 7–16.

[46] Lindsell JA, Klop E (2013) Spatial and temporal variation of carbon stocks in a lowland tropical forest in West Africa. Forest Ecology and Management 289: 10–17.

[47] Magurran AE (2004) Measuring Biological Diversity. Blackwells. 10.1016/j.cub.2021.07.049 34637726

[48] Felde GW, Anderson GP, Cooley TW, Matthew MW, Adler-Golden SM, et al (2003) Analysis of Hyperion data with the FLAASH Atmospheric Correction Algorithm. In Geoscience and Remote Sensing Symposium 2003 IGARSS'03. Proceedings 2003 IEEE International 1: 90–92.

[49] Green AA, Berman M, Switzer P, Craig MD (1988) A transformation for ordering multispectradata in terms of image quality with implications for noise removal. IEEE Transactions on Geoscience and Remote Sensing 26: 65–74.

[50] Tsai F, Philpot W (1998) Derivative analysis of hyperspectral data. Remote Sensing of Environment 66: 41–51.

[51] Gong P, Ru R, Yu B (1997) Conifer species recognition: an exploratory analysis of insitu hyperspectral data. Remote Sensing of Environment 62: 189–200.

[52] Han J, Rundquist DC (1997) Comparison of NIR/RED ratio and first derivative of estimating algal-chlorophyll concentration: a case study in a turbid reservoir. Remote Sensing of Environment 62: 253–261.

[53] Demetriades-Shah TH, Steven MD, Clark J (1990) High resolution derivatives spectra in remote sensing. Remote Sensing of Environment 33: 55–64.

[54] Gamon JA, Penuelas J, Field CB (1992) A narrow-waveband spectral index that tracks diurnal changes in photosynthetic efficiency. Remote Sensing of Environment 41: 35–44.

[55] Sims DA, Gamon JA (2002) Relationships between leaf pigment content and spectral reflectance across a wide range of species, leaf structures and developmental stages. Remote Sensing of Environment 81: 337–354.

[56] Kaufman YJ, D Tanre (1996) Strategy for direct and indirect methods for correcting the aerosol effect on remote sensing: from AVHRR to EOS-MODIS. Remote Sensing of Environment 55: 65–79.

[57] Vogelmann JE, Rock BN, Moss DM (1993) Red edge spectral measurements from sugar maple leaves. International Journal of Remote Sensing 14: 1563–1575.

[58] Gamon JA, Surfus JS (1999) Assessing leaf pigment content and activity with a reflectometer. New Phytologist 143: 105–117.

[59] Sellers PJ (1985) Canopy reflectance, photosynthesis and transpiration. International Journal of Remote Sensing 6: 1335–1372.

[60] Gitelson AA, Merzlyak MN, Chivkunova OB (2001) Optical properties and non-destructive estimation of anthocyanin content in plant leaves. Photochemistry and Photobiology 71: 38–45. 10.1562/0031-8655(2001)074<0038:opaneo>2.0.co;2 11460535

[61] Genuer R, Poggi J-M, Tuleau-Malot C (2010) Variable selection using Random Forests. Pattern Recognition Letters 31: 2225–2236.

[62] Liaw A, Wiener M (2002) Classification and regression by Random Forest. R news 2: 18–22.

[63] Walton JT (2008) Subpixel urban land cover estimation: comparing cubist, andom Forests, and Support Vector Regression. Photogrammetric Engineering & Remote Sensing 74: 1213–1222.

[64] Abdel-Rahman EM, Ahmed FB, Ismail R (2013) Random forest regression and spectral band selection for estimating sugarcane leaf nitrogen concentration using EO-1 Hyperion hyperspectral data. Int J Remote Sens 34: 712–728.

[65] Connor EF, McCoy ED (1979) The statistics and biology of the species-area relationship. American Naturalist 113: 791–833.

[66] Lucas R, Mitchell A, Bunting P (2008) Hyperspectral data for assessing carbon dynamics and biodiversity of forests. In: Hyperspectral Remote Sensing of Tropical and Sub-Tropical Forests. Edited by Kalacska M and Sanchez-Azofeifa GA. CRC Press.

[67] Rocchini D, Vannini A (2010) What's up? Testing spectral heterogeneity vs. NDVI relationship by quantile regression. International Journal of Remote Sensing 31: 2745–2756.

[68] Grime JP (1998) Benefits of plant diversity to ecosystems: immediate, filter and founder effects. J. Ecol 86: 902–910.

[69] Chambers JQ, Asner GP, Morton DC, Anderson LO, Saatchi SS, et al (2007) Regional ecosystem structure and function: ecological insights from remote sensing of tropical forests. Trends in Ecology & Evolution 22: 414–423. 17493704 10.1016/j.tree.2007.05.001

[70] He KS, Rocchini D, Neteler M, Nagendra H (2011) Benefits of hyperspectral remote sensing for tracking plant invasions. Diversity and Distributions 17: 381–392.

[71] Asner GP, Jones MO, Martin RE, Knapp DE, Hughes RF (2008b) Remote sensing of native and invasive species in Hawaiian forests. Remote Sensing of Environment 112: 1912–1926.

[72] Féret JB, Asner GP (2011) Spectroscopic classification of tropical forest species using radiative transfer modeling. Remote Sensing of Environment 115: 2415–2422.

[73] Dalponte M, Bruzzone L, Gianelle D (2008) Fusion of hyperspectral and LIDAR remote sensing data for classification of complex forest areas. IEEE Trans. Geosci. Remote Sens 46: 1416–1427.

[74] Jones TG, Coops NC, Sharma T (2010) Assessing the utility of airborne hyperspectral and LiDAR data for species distribution mapping in the coastal Pacific Northwest, Canada. Remote Sens. Environ 114: 2841–2852.

