# Correction: Biodiversity Mapping in a Tropical West African Forest with Airborne Hyperspectral Data

**DOI:** 10.1371/journal.pone.0105032

**Published:** 2014-08-04

**Authors:** The PLOS ONE Staff

## Notice of Republication

This article was republished on July 10, 2014, to correct an error in the citation. The publisher apologizes for the error. Please download this article again to view the correct version. The originally published, uncorrected article and the republished, corrected article are provided here for reference.

## Supporting Information

File S1 Originally published, uncorrected article.
(PDF)

File S2 Republished, corrected article.
(PDF)
